# Homology modeling of DFG-in FMS-like tyrosine kinase 3 (FLT3) and structure-based virtual screening for inhibitor identification

**DOI:** 10.1038/srep11702

**Published:** 2015-06-29

**Authors:** Yi-Yu Ke, Vivek Kumar Singh, Mohane Selvaraj Coumar, Yung Chang Hsu, Wen-Chieh Wang, Jen-Shin Song, Chun-Hwa Chen, Wen-Hsing Lin, Szu-Huei Wu, John T. A. Hsu, Chuan Shih, Hsing-Pang Hsieh

**Affiliations:** 1 Institute of Biotechnology and Pharmaceutical Research, National Health Research Institutes, 35 Keyan Road, Zhunan, Miaoli County 350, Taiwan, ROC; 2 Centre for Bioinformatics, School of Life Sciences, Pondicherry University, Kalapet, Puducherry 605014, India

## Abstract

The inhibition of FMS-like tyrosine kinase 3 (FLT3) activity using small-molecule inhibitors has emerged as a target-based alternative to traditional chemotherapy for the treatment of acute myeloid leukemia (AML). In this study, we report the use of structure-based virtual screening (SBVS), a computer-aided drug design technique for the identification of new chemotypes for FLT3 inhibition. For this purpose, homology modeling (HM) of the DFG-in FLT3 structure was carried using two template structures, including PDB ID: 1RJB (DFG-out FLT3 kinase domain) and PDB ID: 3LCD (DFG-in CSF-1 kinase domain). The modeled structure was able to correctly identify known DFG-in (SU11248, CEP-701, and PKC-412) and DFG-out (sorafenib, ABT-869 and AC220) FLT3 inhibitors, in docking studies. The modeled structure was then used to carry out SBVS of an HTS library of 125,000 compounds. The top scoring 97 compounds were tested for FLT3 kinase inhibition, and two hits (BPR056, IC_50_ = 2.3 and BPR080, IC_50_ = 10.7 μM) were identified. Molecular dynamics simulation and density functional theory calculation suggest that BPR056 (MW: 325.32; cLogP: 2.48) interacted with FLT3 in a stable manner and could be chemically optimized to realize a drug-like lead in the future.

Acute myeloid leukemia, which is typically referred to as AML, is a hematological malignancy characterized by the abnormal growth of white blood cells, leading to the disruption of normal blood cell production in the bone marrow. It is a rare disease, accounting for only 1.2% of deaths due to cancer in the US^1^. However, the incidence of AML in the older population is higher, and the inherent inability of this population to withstand traditional intensive chemotherapy makes the development of novel drugs for AML essential. Moreover, available treatments for AML, including chemotherapy and allogeneic hematopoietic stem cell (HSC) transplantation, results in a maximum of 5-year survival of only 47% in younger population and 20% in older population^2^.

FMS-like tyrosine kinase 3 (FLT3) is a type III receptor tyrosine kinase with an extracellular ligand binding domain, a transmembrane domain and a cytoplasmic tyrosine kinase domain^3^. It is highly expressed in hematopoietic stem and progenitor cells. The binding of the FLT3 ligand to the extracellular domain leads to the activation of cytoplasmic tyrosine kinase activity, activating downstream cellular signaling that is essential for proliferation. Approximately 23% of AML patients possess an activating internal tandem duplication (ITD) mutation in the juxtamembrane (JM) domain/kinase domain (TK) of FLT3 (FLT3-ITD) and 7% patients possess a point mutation (D835) in the kinase domain (KD)^4^. These mutations makes FLT3 constitutively activated, which leads to the downstream signaling and uncontrolled proliferation characteristic of AML^5^. Hence, the inhibition of FLT3 tyrosine kinase activity, including that of the mutated forms, by small molecules is now recognized as a novel treatment option for AML patients^6^,^7^.

Over the past decade, a number of FLT3 inhibitors have been investigated in clinical trials for the treatment of AML^8^, including sunitinib (SU11248)^9^, lestaurtinib (CEP-701)^10^, midostaurin (PKC-412)^11^, sorafenib^12^, linifanib (ABT-869)^13^ and AC220^14^. These agents competitively inhibit the activity of FLT3 by binding to the ATP binding site of this enzyme. Although all of these agents bind to the ATP binding site, there are subtle differences in their binding modes that are based on the conformation of the conserved DFG (Asp-Phe-Glu) motif in the activation loop. Particularly, the position of the Phe residue of the DFG motif determines the conformation of the activation loop. When the phenyl group of the Phe residue is oriented outside of the ATP binding site, the DFG motif adopts the “in” conformation (DFG-in); alternatively, this motif adopts the “out” conformation if the phenyl group of the Phe residue is oriented inside of the ATP binding site (DFG-out). Inhibitors that bind to the DFG-in conformation are termed type-I inhibitors, and those that bind to the DFG-out conformation are referred to as type–II inhibitors. Type-II inhibitors, in addition to binding to the ATP site, also bind to an additional region termed the “back-pocket” region, which is vacated by the movement of the Phe residue. This back-pocket region is not available for occupation by type-I inhibitors due to the presence of the Phe residue^15^. The active kinase typically adopts the DFG-in conformation, while the inactive enzyme adopts the DFG-out conformation. Based on their preferences for binding to the active or inactive kinase, the known FLT3 inhibitors SU11248, CEP-701, and PKC-412 are classified as type-I inhibitors, while sorafenib, ABT-869 and AC220^14^ are considered type-II inhibitors^16^.

Although both type-I and type-II inhibitors are known to be useful for inhibiting FLT3 enzyme activity, recent studies by Wodicka *et al.*^16^ have shown that type-I inhibitors bind more strongly to the FLT3-ITD-mutated kinase and hence show more promise as an effective treatment for AML. Moreover, Smith *et al.*^5^ have also implied that type-I inhibitors can effectively bind to the active loop mutants D835 and Y842. These findings further emphasize the importance of identifying and developing type-I inhibitors that bind to the FLT3 enzyme in the DFG-in conformation. In this report, we aimed to identify type-I inhibitors through computer-aided drug design (CADD).

The rational design of drugs through structure-based drug design (SBDD) is an effective and efficient tool for discovering drugs and is one of the key techniques of CADD. In SBDD, drugs are designed so that they have shape and charge complementarity to the 3D structure of the target protein. If the 3D structure of the target protein is unknown, homology modeling and associated techniques could be used to predict this structure from its amino acid sequence. This 3D structure could guide both lead optimization and the identification of new leads for the target through the structure-based virtual screening (SBVS) of compound libraries^17^.

To date, only one FLT3 3D structure (PDB ID: 1RJB) is available in the protein data bank, which is in an auto-inhibited DFG-out conformation^18^. However, this 3D structure could not be applied for the SBVS of compound libraries to identify type-I inhibitors. Hence, the 3D structure of FLT3 in the DFG-in conformation was determined using homology modeling based on two template 3D structures, including 1RJB (FLT3, DFG-out conformation) and 3LCD (colony-stimulating factor-1 receptor, CSFR-1, DFG-in conformation). Furthermore, the SBVS of in-house HTS library compounds using this model resulted in the identification of two FLT3 inhibitors with low micromolar IC_50_ values. Finally, docking and molecular dynamics (MD) simulations revealed that both inhibitors bound to the DFG-in FLT3 ATP binding site and stably interacted, suggesting that they are suitable leads for further lead optimization efforts. In addition, we have recently reported the use of this model to guide the design of the dual Aurora kinase A and FLT3 inhibitor BPR5K077^19^. The overall CADD strategy used in this study is shown in Fig 1.

## Results and Discussion

### Construction of DFG-in FLT3 model structure

**Figure 2:**
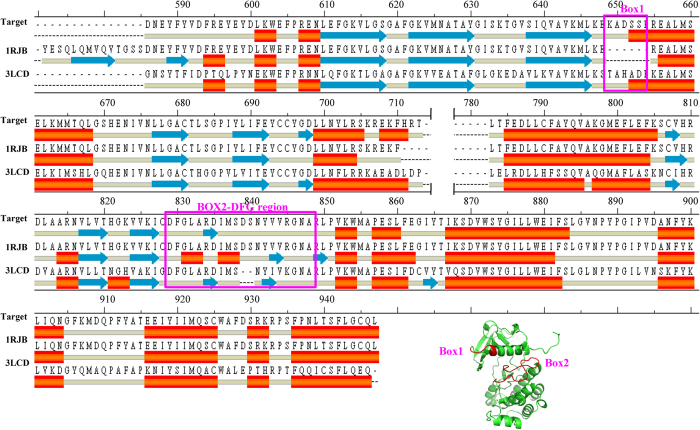
Sequence alignment of the target protein (FLT3) with the template structures 1RJB (DFG-out FLT3 kinase domain) and 3LCD (DFG-in CSF-1 kinase domain). The purple box (Box 1 and Box 2) represents the regions of the template protein 1RJB, which was constructed based on 3LCD atomic coordinates during the homology modeling of DFG-in FLT3.

For modeling the FLT3 structure, NCBI reference sequence: NP_004110.2 was chosen as the target sequence. For the construction of the FLT3 kinase domain in the DFG-in conformation, the DFG-out FLT3 kinase domain 3D structure (PDB ID: 1RJB) was chosen as the template^18^. The 1RJB structure shared a sequence identity of 93% with the target sequence. We planned to use 1RJB as the primary template and to replace the DFG-out motif with a DFG-in motif from a second template structure. For the identification of the second template structure, an NCBI protein-BLAST search was performed against the FLT3 kinase domain 293-amino acid target sequence. The BLAST search identified CSF-1 or the cFMS X-ray structure (PDB ID: 3LCD) in the DFG-in conformation as the appropriate second template. CSF-1 is also a tyrosine kinase receptor, and it was found to share a 62.7% sequence identity with that of FLT3 kinase^20^. The sequence alignment of the two templates (1RJB and 3LCD) against the target FLT3 sequence is shown in Fig 2.

As shown in Fig 2, there are two regions (magenta-colored box) in the target sequence that need to be constructed from the 3LCD structure. The first is the loop region corresponding to the Lys649 to Ser654 residues, which are missing from the 1RJB structure, and the other is the DFG-containing activation loop region residues Asp729 to Ala848. Using the atomic coordinates of 1RJB as the primary template and those of the above-mentioned two regions from the 3LCD structure, the FLT3 DFG-in kinase model was built by Discovery Studio 2.1/MODELER with a medium level of loop optimization.

### Validation of DFG-in FLT3 modeled structure

The quality of the model was evaluated to ensure that it was suitable for carrying out further studies. For this purpose, online quality evaluation tools, including ERRAT^21^, PROCHECK^22^, and Profiles-3D^23^, were used. Prior to starting the actual evaluation process, the active site residues of the DFG-in FLT3 kinase were determined. The identification of active site residue can help to determine whether these regions of the protein are well optimized in the modeled structure.

To define the active site, the coordinates of the backbone Cα atoms of the modeled structure and X-ray crystal structure of human phosphorylase kinase gamma 2 (hPKG2, PDB ID: 2Y7J)^24^ were superimposed using Discovery Studio 2.1/Align structure program. The 2Y7J crystal structure was bound to the kinase inhibitor sunitinib at the active site and was found to share a sequence similarity of 18.9% with that of the modeled FLT3 structure. Because sunitinib is also an inhibitor of FLT3 kinase, the superimposition of the modeled FLT3 with sunitinib-bound hPKG2 helped to define the possible binding site of sunitinib in the DFG-in FLT3-modeled structure. Sunitinib bound hPKG2 was used to define the active site, as sunitinib is already an approved drug. The superimposed structures along with the binding of sunitinib to the active site are shown in supplementary Fig S1.

Once the active site residues were identified, the overall quality factor of the modeled structure was assessed using ERRAT program, which determines the false statistics of bad non-bonded interactions within the modeled structure. The ERRAT score is expressed as the percentage of residues in which the calculated error value falls below the 95% rejection limit. A high-resolution X-ray structure will be generally graded with an ERRAT score of 95% or higher. However, low-resolution (2.5–3.0 Å) structures have ERRAT scores of approximately 91%. The overall quality factor of the DFG-in FLT3 modeled structure was 84.53% (Supplementary Fig S2).

Because ERRAT analysis of the modeled DFG-in FLT3 structure showed that its quality was less than optimal, it was subjected to 500,000 steps of MD simulations with the GVMB implicit solvent model for quality improvement by allowing the protein to relax the bad contacts^25^. Analysis of the MD-simulated structure showed that the ERRAT score was increased to 96.03% (Fig S2). Particularly, MD simulation helps to fix the bad contacts at the active site of the protein. The black arrows in Fig S2 show that the residues (Val641-Lys644, Asp811-Val819, and Lys826-Gly831) within 7 Å in distance from sunitinib were further refined following the MD simulation step.

Additional analysis of the structure using the Profile-3D Verify score showed that MD simulation also increased this score to 118.58 from 107.22. The expected Verify high score for this model is 133.30, and the expected Verify low score is 59.99. Models with a Verify score that is closer to the expected high score are considered optimal^26^. Furthermore, MD simulation improved the scores for both regions I and II, which contain the active site residues (Val624, Val641-Lys644, Leu818, and Cys828), implying that the MD simulation corrected the errors in these residues (Supplementary Fig S3). Moreover, a Ramachandran plot of the MD-simulated structure showed that 90% of the residues were in the most favored region, and only 0.8% were in the disallowed region (Supplementary Fig S4). Typically, >90% of residues should be in the most favored region for a modeled structure to be considered suitable for further study^22^. Quality evaluation using the online tools ERRAT, PROCHECK, and Profiles-3D suggested that the MD-simulated DFG-in FLT3-modeled structure was of suitable quality for further analysis.

### Effect of internal tandem duplication (ITD) insert to the juxtamemberane and tyrosine kianse domain of FLT3-DFG-in modeled structure

The internal tandem duplication (ITD) and D835 point mutation play an important role in AML development. The ITD insertion is possible either at the JM (69.5%) or at TK-1 domain (30.5%)^27^. In order to ascertain what effect ITD insertion could have on the inhibitor binding in FLT3 DFG-in model structure, we generated the binding site using Discovery Studio 2.1 Program (Fig S5, supporting information). Depending on the place of ITD insert, the inhibitor binding will be affected. From the figure it is clear that ITD insert to JM domain is further away from the inhibitor binding site to influence the inhibitor binding. However, ITD insert at the TK domain is proximal to the ligand binding site and may influence the inhibitor binding. This is in agreement with the observation made by Kayser *et al.*^27^, that ITD in the TK-1 domain could result in resistance to TK inhibitors. In addition, the D835 mutation is away from the inhibitor binding site and could have minimal effect on inhibitor binding. Recently, Smith *et al.*^5^, had shown that type-I inhibitors can effectively bind to the active loop mutants D835. Moreover, Wodicka *et al.*^16^, also suggest that type-I inhibitors bind more strongly to the FLT3-ITD-mutated kinase, which is in line with our observations.

### Comparison of binding modes of known inhibitors to DFG-in and DFG-out FLT3 structures

**Figure 3:**
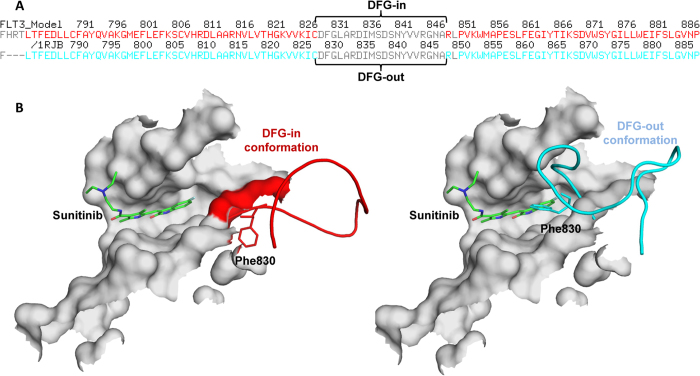
(**A**) The sequence alignment of the target protein (FLT3) with the template structure (PDB ID: 1RJB) in the DFG loop region. (**B**) Comparison of the DFG-in (red, homology-modeled structure) and DFG-out FLT3 structures (cyan, X-ray structure PDB ID: 1RJB). Sunitinib bound to the DFG-out FLT3 kinase showed a steric clash with the Phe830 residue.

Once the DFG-in FLT3-modeled structure was refined using MD simulation and was ready for further analysis, the structural differences between the DFG-in and DFG-out conformations were compared by superimposition of the FLT3-modeled (DFG-in) structure on the 1RJB (DFG-out) template structure (Fig. 3). This superimposition clearly showed that the activation loop in the DFG-in structure flipped away from the active site, leading to the placement of the Phe830 group away from this site, while in the DFG-out structure, the activation loop flipped over to the active site, resulting in the placement of Phe830 in the active site. The different arrangement of the activation loop, particularly the placement of the Phe830 group, has important implications for the binding of different types of inhibitors to FLT3 kinase. For example, the superimposition of sunitinib coordinates from 2Y7J onto FLT3 showed that there was a steric clash between the ligand and the Phe830 residue of the DFG-out motif, but no steric clash between the ligand and the Phe830 residue was observed in the DFG-in-modeled structure. Sunitinib is a known type-I inhibitor that is able to bind to FLT3 in the DFG-in conformation much more strongly than the DFG-out conformation.

To further validate the above observation that sunitinib could have differential binding to the DFG-in and DFG-out confirmations of FLT3, we investigated the binding modes of other known FLT3 inhibitors to these two structures. Wodicka *et al.*^16^ have distinguished several FLT3 inhibitors as either type-I or type-II. Type-I FLT3 inhibitors include CEP-701^10^, PKC-412^11^, and SU11248^9^. Type-II FLT3 inhibitors include sorafenib^12^, ABT869^13^, and AC220^14^. These clinical trial compounds were docked to the FLT3 DFG-in and DFG-out structures using Dock 6.0^28^, and docking scores and binding energies were calculated (Table 1). The type-I inhibitors were observed to have higher docking scores than the type-II inhibitors for the active site of the DFG-in structure. In contrast, the type-II inhibitors had better docking scores for the DFG-out structure. Similarly, binding energy calculation also revealed the better binding of the type-I inhibitors to the DFG-in structure (−34.21 to −25.52) than to the DFG-out structure (17.62 to 27.90). However, the exact opposite results were observed for the type-II inhibitors, which possessed better binding energies for the DFG-out structure (−13.64 to −7.89) compared with the DFG-in structure (−0.61 to −7.06). These docking studies revealed that the modeled DFG-in FLT3 structure was able to distinguish between the type-I and type-II inhibitors based on the docking scores and binding energies. Hence, the DFG-in FLT3-modeled structure may be used to identify new type-I FLT3 inhibitors.

### Structure-based virtual screening to identify new DFG-in FLT3 inhibitors

Next, the DFG-in-modeled structure was used to perform virtual screening (VS) of an in-house compound database (125,000 compounds; obtained from ChemDiversity, http://www.chemdiv.com) using Dock 6.0. We have successfully used this chemically diverse library both for high-throughput screening as well as VS campaign for different targets, including, for influenza^29^,^30^, EGFR kinase^31^, aurora kinase^32^,^33^, tubulin target^34^ etc. The screening parameters were the same as those used in the docking study of the clinical trial drugs. The database compounds were ranked based on their Dock score and a total of 5641 were found to have higher Dock score than sunitinb (Dock score −300).These compounds were subjected to second round of virtual screening by using DS/Ligfit. The Ligfit score of sunitinib (−88.56) was set as a threshold to rank these compounds. A total of 97 compounds were found to have better LigFit score than sunitinib and were selected for biochemical testing to assess FLT3 inhibition at 10 μM concentration (Supplementary Table S1). Of the 97 compounds tested, two compounds, BPR056 and BPR080, showed more than 40% inhibition at a 10 μM concentration. Further evaluation showed that these two compounds are low-micromolar inhibitors of FLT3, with IC_50_ values of 2.3 μM and 10.7 μM, respectively (Table 2, and Supplementary Fig S6).

In order to assess the performance of the virtual screening process^35^, we calculated the hit rate (only considers number of actives and number of tested compounds) and enrichment factor (also takes into consideration number of database compounds) and found to be 2.06% and 1289, respectively. Comparison of these two performance parameters with that for five recently reported virtual screening studies shows that the performance of the current method is comparable (Supplementary table S2).

It should also be noted that the hits (BPR056, pyridine derivative; BPR080, chromone derivative) identified in this study possess low micromolar inhibition and chemically distinct from our previously reported FLT3 inhibitors (pyrazole and furanopyrimidine derivatives)^19^,^36^,^37^. It is common in drug discovery programs to develop multiple series of compounds in parallel to explore activity profiles, physico-chemical properties and toxicity profile, so that the most suitable compounds can be taken up further for development. Hence, we explored the suitability of the two hits (BPR056 and BPR080) for further development.

### Binding mode analysis of hits in DFG-in FLT3 structure

**Figure 4:**
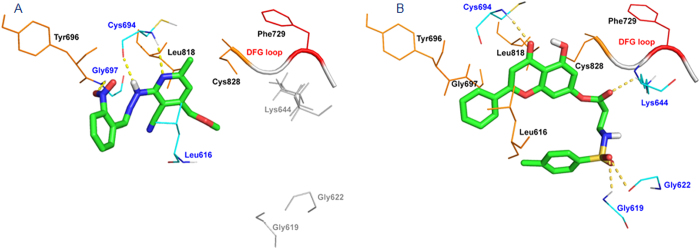
Docking poses of the two hits identified from the VS in the DFG-in FLT3-modeled structure. (**A**) Hit BPR056 (green). (**B**) Hit BPR080 (green). Hydrogen-bonding residues are shown in blue, hydrophobic residues in orange and the DFG-in motif in red.

To understand the binding modes of these two hits in the modeled structure, which may aid in the design of better FLT3 inhibitors, the docking interactions were analyzed in detail, which are shown in Fig 4. Both hits bound at the ATP binding site by forming H-bonds with the hinge-region Cys694 residue. A hinge-region H-bond interaction between an inhibitor and kinase is essential for inhibitory activity and has been observed in several inhibitor–kinase complex structures^38^. In BPR056, the NH of the imino group formed an H-bond with the backbone carbonyl group of the Cys694 residue, and the nitrogen atom of the pyridyl ring formed a second H-bond with the NH group of the Cys694 residue (Fig. 4A). BPR056 also interacted with the backbone NH of the Tyr696 hinge-region residue through the oxygen atom of the NO_2_ group. The Tyr696, Leu818, and Cys828 residues participated in hydrophobic interactions with BPR056. In the case of BPR080, the chroman-4-one carbonyl group formed one H-bond with the back-bone NH group of the Cys694 residue (Fig. 4B). In addition to the hinge region interaction, BPR080 formed H-bonds with the Gly619, Gly622, and Lys644 residues. It also created hydrophobic contacts with the Leu616, Tyr696, Gly697, Leu818, and Cys828 residues. In addition, the binding energies of both hits in the DFG-in and DFG-out FLT3 structures were calculated and found to be −23.40 Kcal/mol (BPR056) and −22.92 Kcal/mol (BPR080), and 16.41 Kcal/mol (BPR056) and 19.82 Kcal/mol (BPR080), respectively. This indicates that both hits were able to bind to the DFG-in-modeled structure better than to the DFG-out structure in a manner similar to those of known DFG-in binding inhibitors.

### Density functional theory analysis

**Figure 5:**
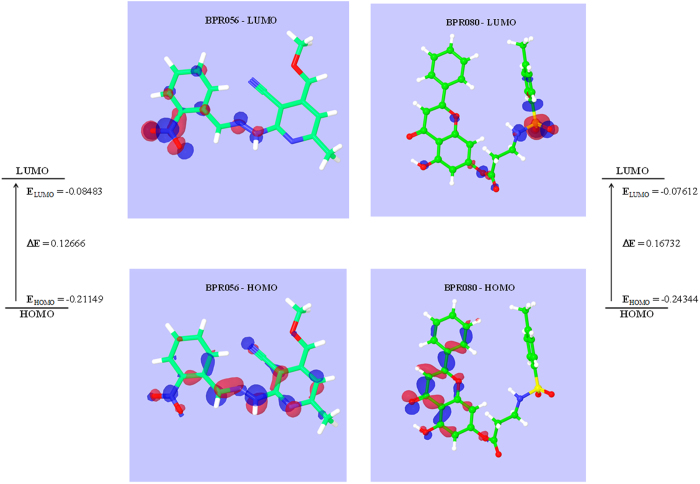
Molecular orbital (HOMO and LUMO) diagram, energies and energy gap for BPR056 and BPR080.

Density functional theory (DFT) is a promising quantum mechanical approach to provide an accurate description of electronic and structural properties of small molecules^39^. The orbital energies were calculated by DFT to provide information about electronic distribution of the two potential inhibitors identified from virtual screening. Information about the electronic distribution could provide insight into the protein - ligand interaction and could be useful to rationalize the inhibition potential of the compounds. The highest occupied and lowest unoccupied molecular orbitals (HOMO and LUMO) and their energies were computed to locate the high and low electron density regions in both inhibitors and are shown in Fig 5. Localization of HOMO and LUMO orbitals in a molecule are important, as electrons from the HOMO orbitals are the most free to participate in a reactions and both are responsible for the charge transfer in a chemical reactions^40^. The higher energy value for HOMO implies a strong reactivity potential; while a smaller energy gap between HOMO and LUMO often shows direct correlation with reactivity^41^. BPR056 shows a lower energy gap between HOMO and LUMO, as compared to BPR080. This implies a better reactivity and hence better inhibition potential for BPR056, as observed in FLT3 kinase inhibition assay. The molecular orbital surface diagram shows that the NH group of the imino function in BPR056 and CO group of the chroman-4-one ring in BPR080 are high electron density region in HOMO and corresponds to functional groups interacting with the important hinge region residue Cys694 of FLT3 kinase as discussed above. Thus the DFT calculations performed here further substantiate our findings in molecular docking and in *in vitro* enzyme inhibition studies.

### Molecular dynamics simulation study

**Figure 6:**
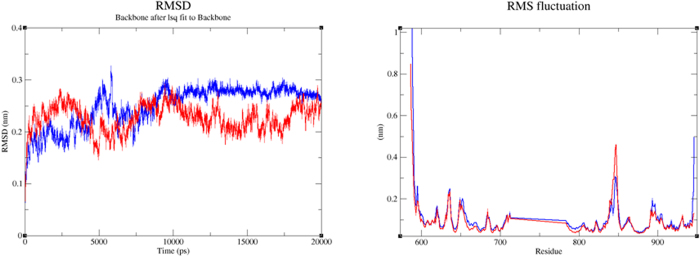
Molecular dynamics simulation (20 ns) of the DFG-in FLT3 complexed with the two hits identified in the VS. (**A**) Protein backbone root mean square deviation (RMSD) graph for BPR056 (blue) and BPR0080 (red). (**B**) Protein αC atom root mean square fluctuation (RMSF) graph for the BPR056 (blue) and BPR080 (red) complex.

In addition, the DFG-in FLT3-inhibitor (BPR056 and BPR080) complex structures were subjected to 20 ns of MD simulation using GROMACS v4.6.5 package and analyzed to determine the stability of the predicted interactions. The RMSD of the protein backbone and the RMSF of the αC atoms of the amino acids during the simulation are shown in Fig 6. The H-bond that formed between the protein and ligand and the RMSD of the ligand during the simulation are shown in supplementary Fig S7. A comparison of the RMSDs of the protein backbones in both cases showed that BPR056 reached equilibrium conditions very early and remained stable after 10 ns, indicating that the FLT-3–BPR056 complex was much more stable during the simulation. Further, analysis of the RMSF graph showed that the major differences between the two complexes occurred in the loop region residue Asn847, which showed more fluctuation in the FLT-3-BPR080 complex.

The average protein-ligand H-bond formation during the simulation period for the FLT-3-BPR056 complex was 0.78, and it was 0.98 for the FLT-3-BPR080 complex. BPR056 established H-bonds between Lys614, Asn626, Tyr693, Cys694, Gly697 and Asp698 and the hinge region residue Cys694, forming the most stable H-bond (68% existence) observed during the simulation period. Similarly, BPR080 established H-bonds between Ser618, Gly619, Val624, Glu692 Cys694, Asn816 and Asp829 and the hinge region residue Cys694, forming the most stable H-bond (93% existence) observed during the simulation period. The above analysis of the protein and ligand trajectories showed that both of the hits were bound to the ATP binding site throughout the simulation period. In particular, BPR056 (Ml wt: 325.32; cLogP: 2.48) may be a suitable initial lead for further modifications.

## Conclusion

Known FLT3 inhibitors bind to the kinase domains of this enzyme in either DFG-in or DFG-out conformation. For these confirmations, the Phe group of the DFG motif either projects into the ATP binding site (DFG-out) or away from it (DFG-in), leading to the differential binding of inhibitors at this site. Recently, inhibitors that are able to bind to the DFG-in conformation of FLT3 have also been reported that inhibit the mutant forms of the kinase; thus, they may be more useful for the treatment of AML. To harness the power of SBDD for the identification of novel DFG-in-specific FLT3 inhibitors, homology modeling was initially carried out to construct the DFG-in FLT3 3D structure. Then, the DFG-in FLT3-modeled structure was refined by MD simulation and validated using the online servers ERRAT, PROCHECK, and Profile 3D. The modeled structure was further validated by assessing its ability to correctly identify the known DFG-in (SU11248, CEP-701, and PKC-412) and DFG-out (sorafenib, ABT-869 and AC220) FLT3 inhibitors based on their docking scores.

Second, the DFG-in FLT3-modeled structure was used to perform SBVS of an in-house HTS database of 125,000 compounds. The top scoring 97 compounds were biochemically evaluated for FLT3 inhibition at a 10 μM concentration, and two hits were identified, including BPR056 (IC_50_ = 2.3 μM) and BPR080 (IC_50_ = 10.7 μM). Detailed docking, DFT calculations and 20-ns MD simulation of the new hits in the DFG-in FLT3-modeled structure revealed that BPR056 (Ml wt: 325.32; cLogP: 2.48) formed a stable interaction with the target and that it may be a suitable lead for AML treatment. Additionally, the modeled FLT3 structure allowed for the design and development of the dual Aurora kinase A and FLT3 inhibitor BPR5K077 (IC_50_ = 15 and 67 nM, respectively). In conclusion, the FLT3 DFG-in-modeled structure reported here may help in the identification and development of novel drugs for AML treatment.

## Material and Methods

### Homology modeling

The FLT3 kinase domain, which is 293 amino acids in length, was chosen as the target sequence (NCBI reference sequence: NP_004110.2) to be modeled. The amino acids from 1-585, 711-782, and 948-993 of NP_004110.2 were not used in the model generation. To model the FLT3 structure in the DFG-in conformation, two template structures were chosen. The first template was the X-ray structure of FLT3 in the DFG-out (PDB ID: 1RJB)^18^ conformation, which shared a sequence identity of 93% (the sequence identity was calculated by the NCBI protein-BLAST program, http://blast.st-va.ncbi.nlm.nih.gov/Blast.cgi) with the target sequence. The other template was the DFG-in colony-stimulating factor-1 receptor (CSF-1) X-ray structure (PDB ID: 3LCD), which shared a sequence identity of 63% with the target protein^20^,^42^. CSF-1 is a receptor tyrosine kinase. To construct the protein model for the target sequence, Discovery Studio 2.1 (DS 2.1)/Build Homology models program (Accelrys, Inc., San Diego, CA) was used. Sequences of the 3LCD and 1RJB structures were aligned against the target sequence to identify the matched regions. Based on the atomic coordinates of the matched regions of the template structures, the structure of the target sequence was constructed, followed by loop refinement using the DOPE (Discrete Optimized Protein Energy) method with DS2.1 program^43^.

### Structure refinement and validation

The refinement of the modeled structure to relax the loops and bad contacts was carried out by subjecting the modeled structure to a series of MD steps using the steepest descent and conjugate gradient method with the CHARMm force field^44^. The protein model was initially refined by 500,000 steps of MD simulation with a time step of 1 fs at a constant temperature of 300 K. The simulation was carried out in a Generalized Born with Molecular Volume (GBMV) implicit solvent modeling environment^45^. Both the built and refined protein models were verified using PROCHECK^22^, Profiles-3D^23^, and ERRAT^21^ online servers. PROCHECK program helps to visualize the backbone dihedral angles ψ against φ of the amino acid residues in the protein structure by constructing a Ramachandran plot, and it aids in the quality assessment of the modeled structure. Profiles-3D program helps to evaluate the compatibility of the amino acid sequence with the 3D structure by transforming this structure to a 1D representation, which is called the 3D profile, and calculating a Verify score. ERRAT program helps to determine the false statistics of bad non-bonded interactions within the modeled structure.

### Virtual screening

The binding pocket in the modeled structure was identified by superimposing the modeled DFG-in FLT3 structure onto the X-ray structure of phosphorylase B kinase (DFG-in structure), which was complexed with the ligand sunitinib (PDB ID: 2Y7J, Fig S1). The position of sunitinib at the active site of 2Y7J was used to define the active site of the DFG-in FLT3-modeled structure. A two-stage docking protocol was performed to identify new hits for the DFG-in FLT3-modeled structure using Dock 6.0 and DS2.1/ligFit^28^,^46^. For the docking study, the grid space was set to 0.1 Å, and the energy cutoff distance was 5 Å. The docking parameters were fine-tuned by calculating the root-mean-square-deviation (RMSD) of the X-ray conformation and the docked conformation generated for sunitinib, which was approximately 1.5 Å (Supplementary Fig S8 and Table S3). Then, the fine-tuned docking parameters were applied for the virtual screening (VS) of the in-house HTS library (125,000 compounds) to carry out the first round of screening. The second round of screening was conducted using the DS/Ligfit program with the CHARMm force field^47^.

### Binding energy calculation

The binding energy calculations were performed by DS 2.1/Calculate binding energies program. The 1000-step Smart minimizer method in the GBMV^48^ implicit solvent environment was used with the default parameter values before calculating the binding energies.

### DFT calculations of the hits

For the Density functional theory (DFT) calculations, both the inhibitor poses were imported to Maestro 9.2 of Schrodinger and the Jaguar module^49^ was used to optimize the molecules using functional B3LYP with 6-3IG** basic set^50^. Subsequently, single point energy, molecular orbital surface and electrostatic potential charges were computed.

### Molecular dynamics simulation of the hits

The MD simulations were carried out using GROMACS v4.6.5^51^,^52^ with the AMBER force field amber99sb-ildn at standard temperature (300 K) and pressure (1 bar) using the Berendsen coupling method. All of the coordinates and ligand topologies were generated using the antechamber program of Amber11^53^. The LINCS algorithm was used to constrain the hydrogen bond lengths. The time step was maintained at 2 fs for the simulation. A cut-off distance of 10 Å was used for all short-range non-bonded interactions, and 12 Å Fourier grid spacing was used for the PME method for long-range electrostatics. A two-step energy minimization was performed using steepest descent and conjugate gradient algorithms to converge the system up to 10 kJ mol^−1^ nm^−1^. The NVT and NPT steps were run for 450 ps, and the final production run was performed for 20 ns.

### Biochemical evaluation

The recombinant GST-FLT3 (residues Y567–S993) proteins were expressed and the FLT3 Kinase-Glo kinase assay was performed as reported in our earlier study^37^.

## Additional Information

**How to cite this article**: Ke, Y.-Y. *et al.* Homology modeling of DFG-in FMS-like tyrosine kinase 3 (FLT3) and structure-based virtual screening for inhibitor identification. *Sci. Rep.* **5**, 11702; doi: 10.1038/srep11702 (2015).

## Supplementary Material

Supplementary Information

## Figures and Tables

**Figure 1:**
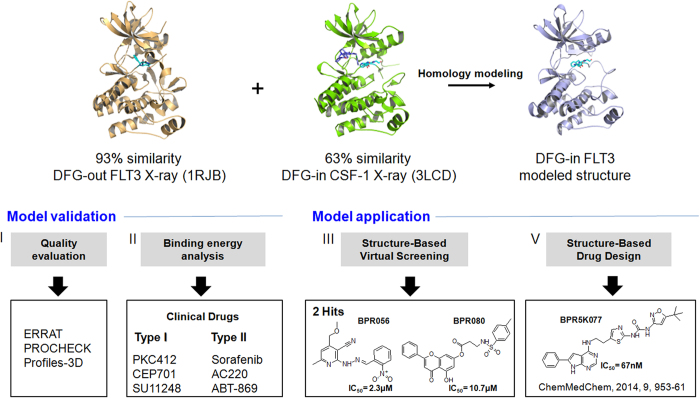
Computer-aided drug design (CADD) strategy for FLT3 inhibitor identification.

**Table 1:** Predicted binding energy of clinical trial compounds in DFG-in and DFG-out FLT3 structure.

					

**Table 2:** FLT3 kinase inhibition profiles, docking score and binding energies of the hits identified from the in-house HTS database. PKC412 and sorafenib are used as reference compounds for the comparison.

					
