# Supplementary material for: Homology modeling of DFG-in FMS-like tyrosine kinase 3 (FLT3) and structure-based virtual screening for inhibitor identification
Source: Sci Rep. 2015 Jun 29;5:11702. doi: 10.1038/srep11702 (PMC4483777; doi:10.1038/srep11702)
Supplement: Supplementary Information [file srep11702-s1.pdf]

Supplementary information

Homology modeling of DFG-in FMS-like tyrosine kinase 3 (FLT3) and structure-based virtual screening for inhibitor identification

Yi-Yu Ke,[†] Vivek Kumar Singh,[⊥] Mohane Selvaraj Coumar,[⊥] Yung Chang Hsu,[†] Wen-Chieh Wang,[†] Jen-Shin Song,[†] Chun-Hwa Chen,[†] Wen-Hsing Lin,[†] Szu-Huei Wu,[†] John T. A. Hsu,[†] Chuan Shih,[†] and Hsing-Pang Hsieh[†,*]

[†] Institute of Biotechnology and Pharmaceutical Research, National Health Research Institutes, 35 Keyan Road, Zhunan, Miaoli County 350, Taiwan, ROC.

[⊥] Centre for Bioinformatics, School of Life Sciences, Pondicherry University, Kalapet, Puducherry 605014, India

[*]To whom correspondence should be addressed: Phone, +886-37-246-166 ext. 35708; fax, +886-37-586-456; e-mail, hphsieh@nhri.org.tw

Contents

A. Figures

Figure S1. Structure alignment of the homology-modeled and DFG-in X-ray structure..........**S3**

Figure S2. Homology model structure validated by ERRAT server…………………………..**S4**

Figure S3. Homology model structure validated by Profile-3D server……………………….**S4**

Figure S4. Homology model structure validated by Ramachandran plot……………….……**S5**

Figure S5. Effect of ITD insert and D835 mutation to FLT3 DFG-in modeled structure….....**S6**

Figure S6 The IC_{50} determination dose-response curve of BPR056 and BPR080……………**S7**

Figure S7 Molecular dynamics simulation of DFG-in FLT3 in complex with two hits (H-bond)……………………………………………………………………………**S7**.

Figure S8. Aligned structures of the original X-ray ligand and docked ligand……..………**S8**

B. Tables

Table S1. The percent FLT3 inhibition at 10 μM for screened compounds…………………...**S9**

Table S2. Comparison of enrichment factor (EF) of our study with 5 recently reported virtual screening experiments.……………………………………………………...**S17**

Table S3. Docking parameters used for the study……………………………………………..**S18**

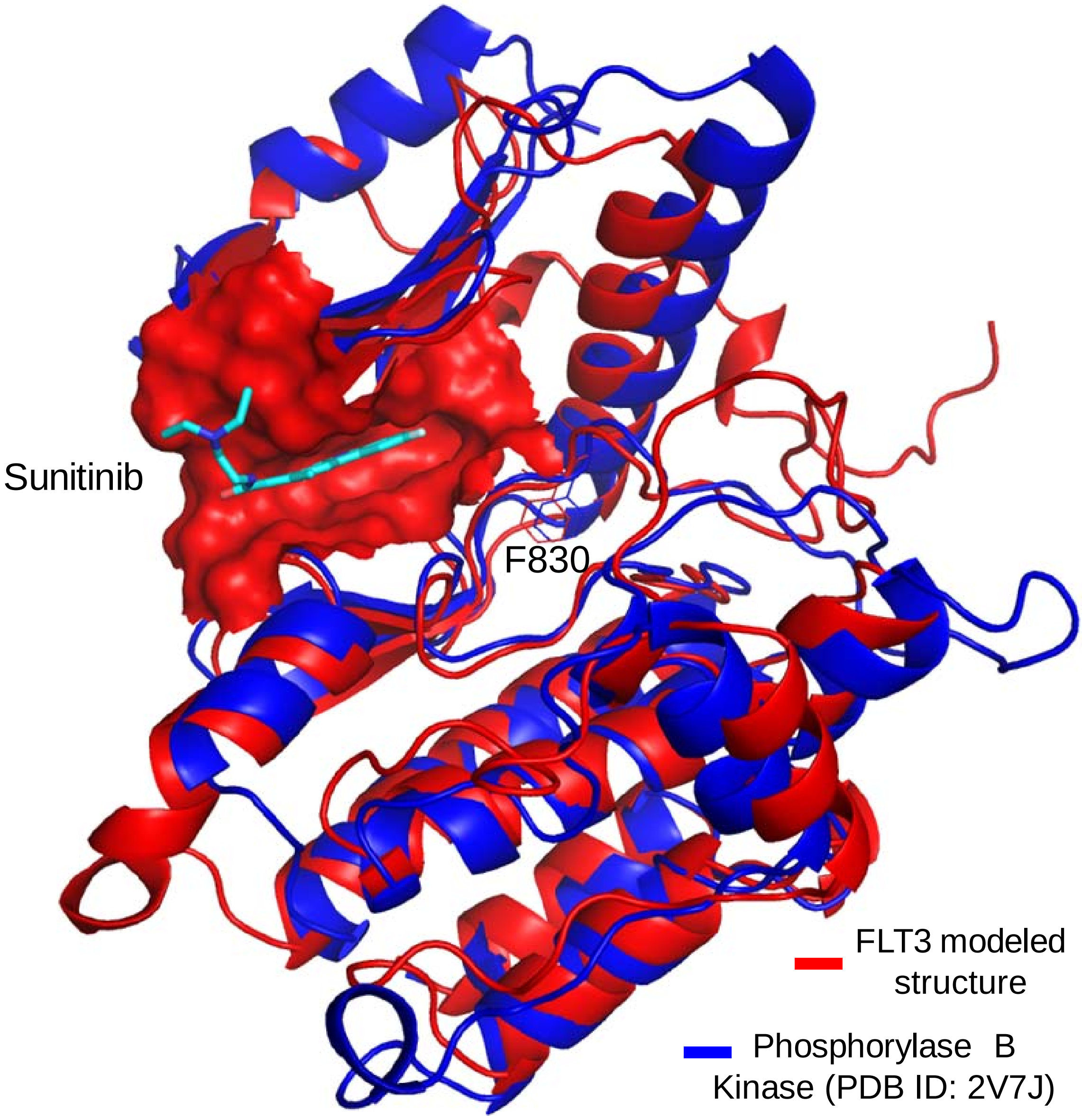

Figure S1. 3D structure alignment of the homology-modeled DFG-in FLT3 structure to the X-ray structure of phosphorylase B kinase (PDB ID: 2Y7J) bound to the ligand sunitinib.

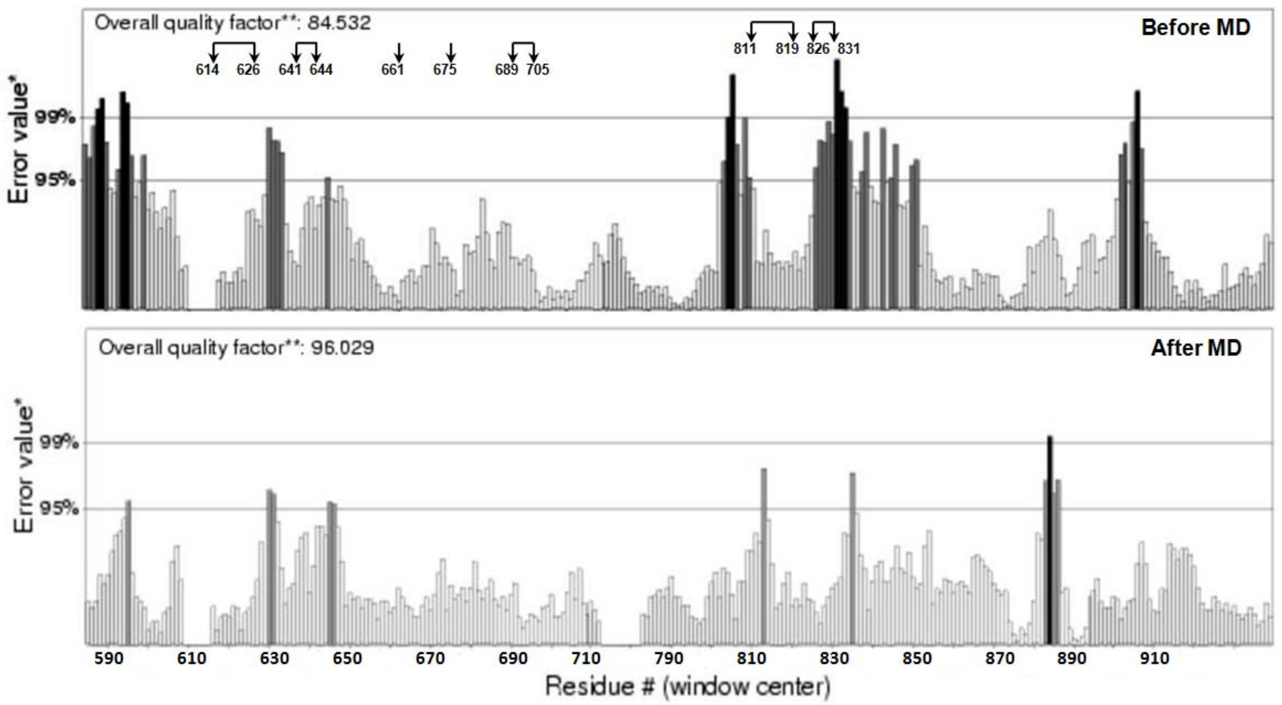

Figure S2. Assessment of the DFG-in FLT3-modeled structure by ERRAT server. The arrows represent the active site residues in the protein model. The overall quality factor of the modeled structure improved after MD simulation.

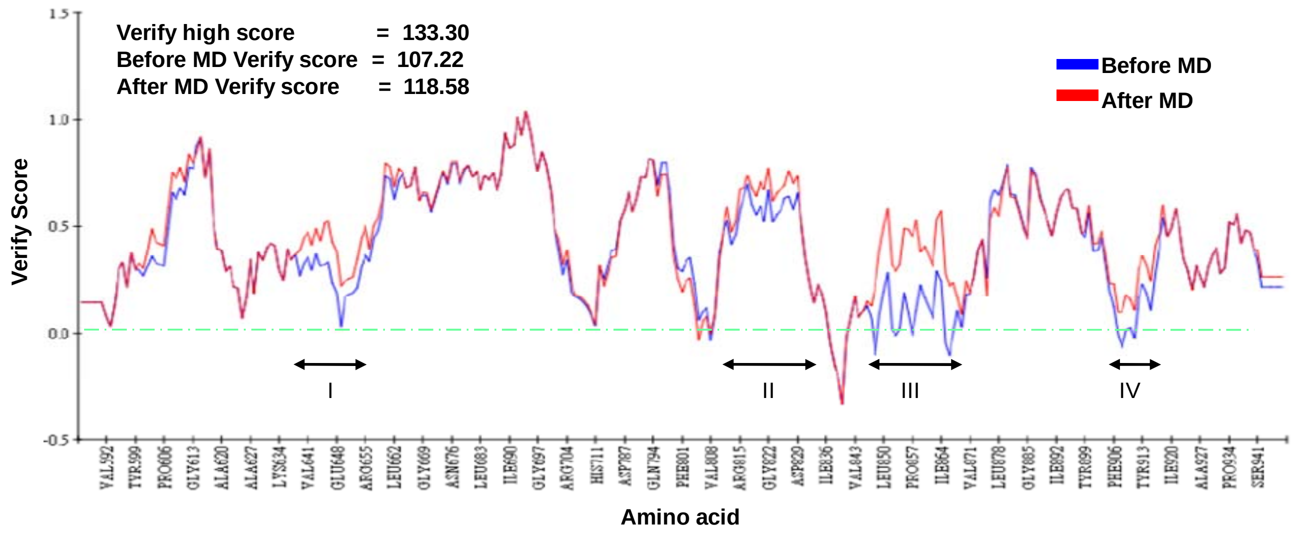

Figure S3. Assessment of the DFG-in FLT3-modeled structure by Profile-3D server. The arrowed regions represent the active site residues in the protein model. The Verify score improved after MD simulation of the modeled structure.

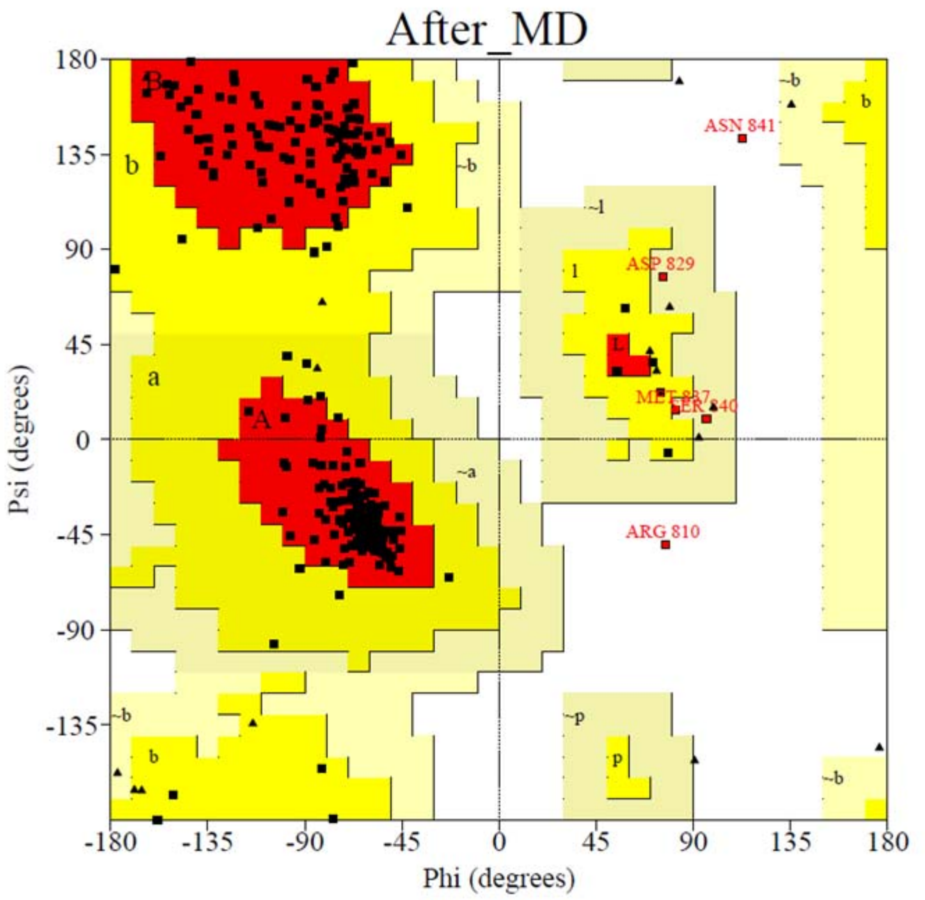

Plot statistics

Residues in most favoured regions [A,B,L]	235	90.0%
Residues in additional allowed regions [a,b,l,p]	21	8.0%
Residues in generously allowed regions [~a,~b,~l,~p]	3	1.1%
Residues in disallowed regions	2	0.8%
	----	------
Number of non-glycine and non-proline residues	261	100.0%
Number of end-residues (excl. Gly and Pro)	2	
Number of glycine residues (shown as triangles)	20	
Number of proline residues	10	

Total number of residues	293	

Based on an analysis of 118 structures of resolution of at least 2.0 Angstroms and R-factor no greater than 20%, a good quality model would be expected to have over 90% in the most favoured regions.

Figure S4. Ramachandran plot of the homology modeled DFG-in FLT3 structure after MD simulation.

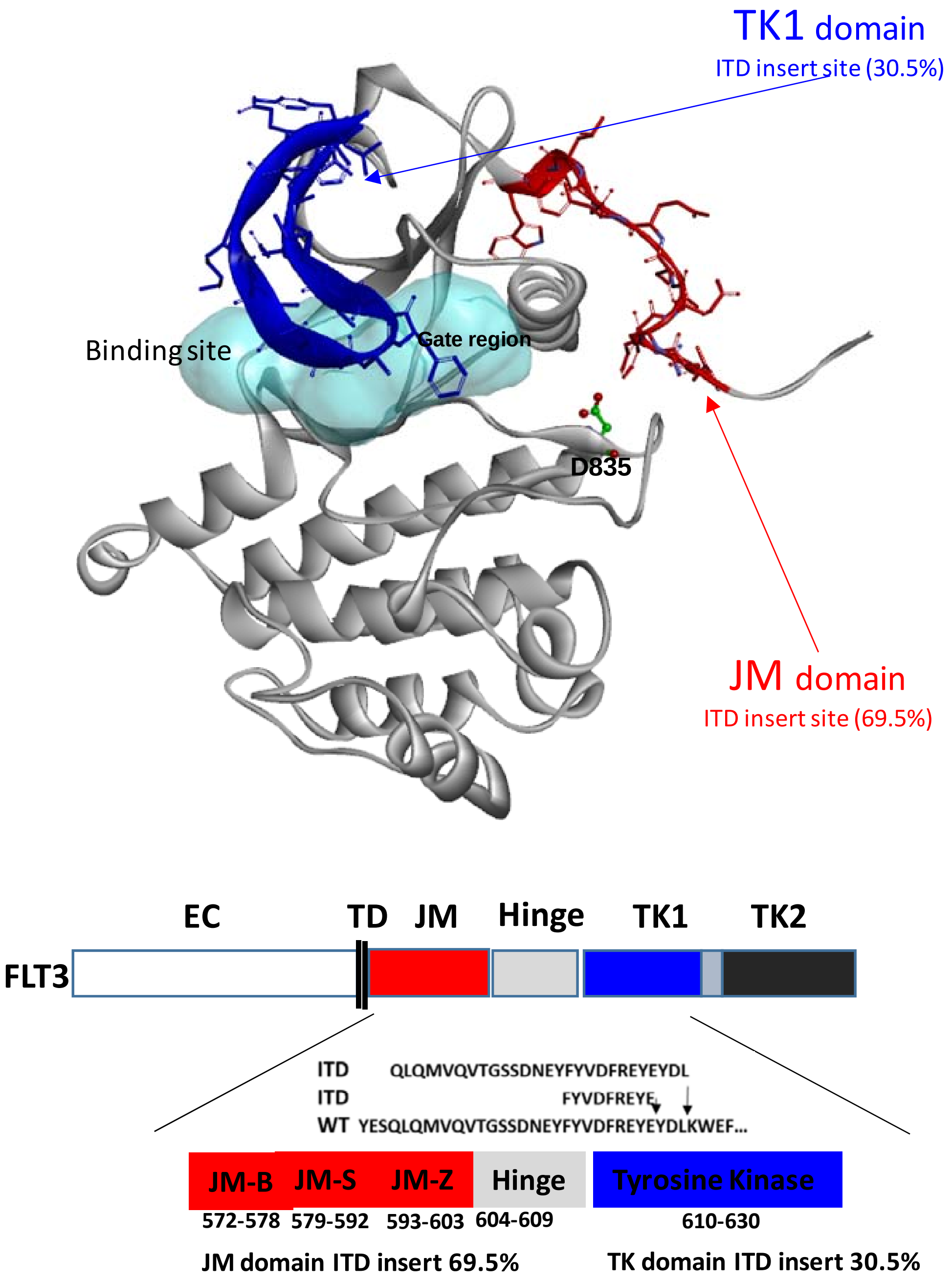

Figure S5. FLT3 (DFG-in) ITD insert site and D835 mutation location. Tyrosine kinase domain-1 (TK1, blue color); juxtamembranne domain (JM, red color) and ligand binding site (blue transparent surface).

FLT3 kinase assay		2013/5/27	Z=	0.69
		windows=	5.4	
µM	CSV0A027968	CSV0A034160	µM	sorafenib
30	61.8%	77.5%	2	92.3%
20	67.2%	98.8%	0.8	103.8%
15	60.4%	108.2%	0.32	82.7%
10	42.4%	79.9%	0.128	60.0%
5	18.3%	66.2%	0.051	33.5%
2.5	9.3%	47.9%	0.020	24.3%
1.25	1.4%	24.7%	0.008	10.1%
0.625	0.4%	16.5%	0.003	8.4%
IC50(µM)	10.7	2.3		0.102

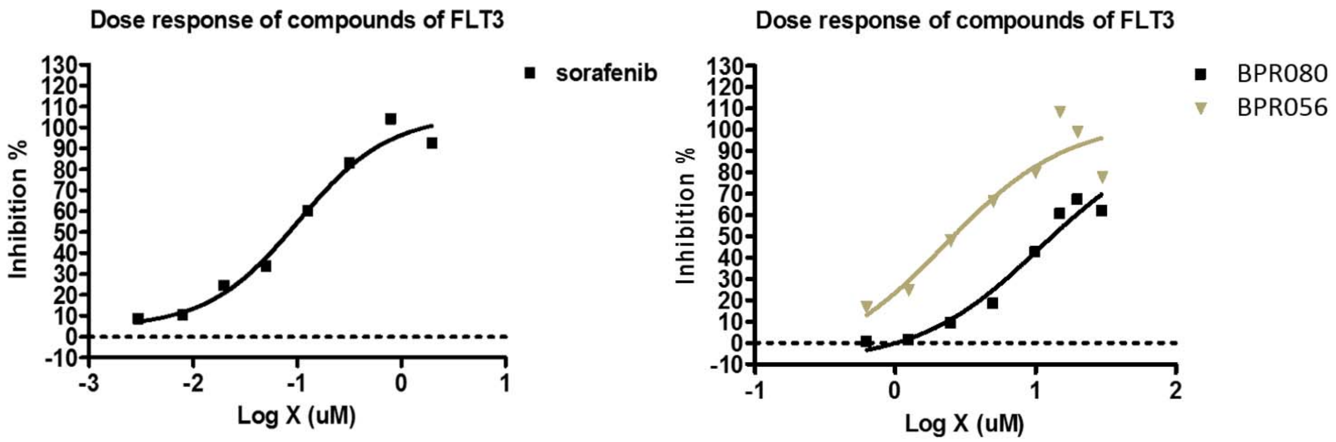

Figure S6. The IC_{50} determination dose-reponse curve of BPR056 and BPR080, along with sorafenib.

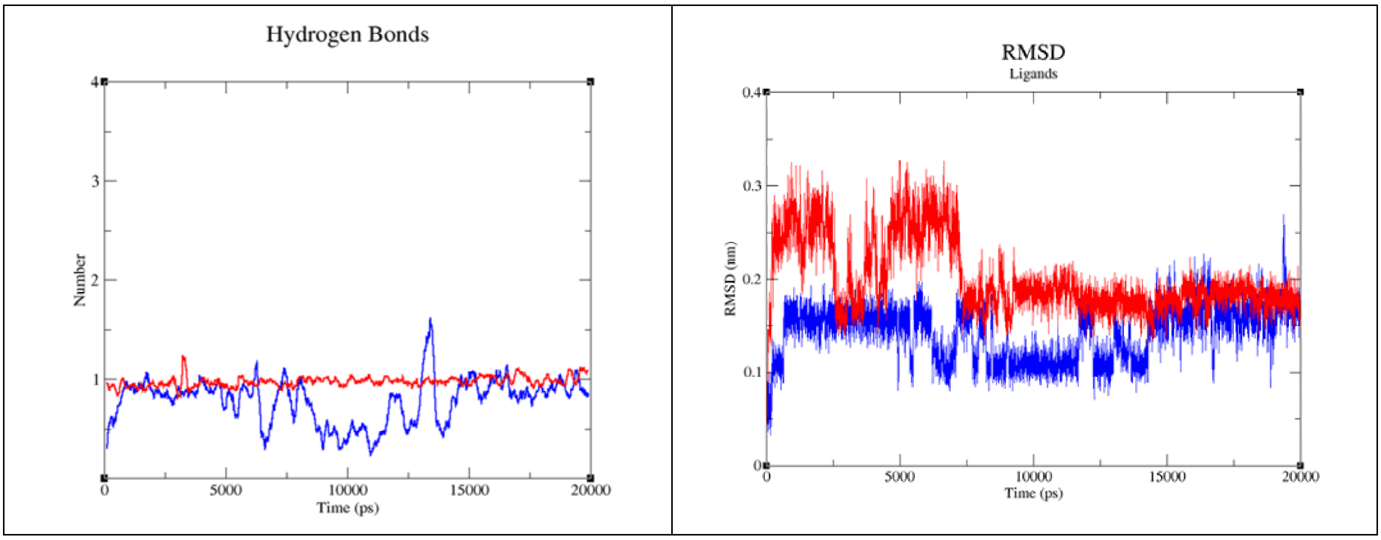

Figure S7. Molecular dynamics simulation (20 ns) of DFG-in FLT3 in complex with two hits identified from VS. (A) H-bond graph for BPR056 (blue) and BPR0080 (red). (B) Ligand root mean square deviation (RMSD) graph for BPR056 (blue) and BPR080 (red).

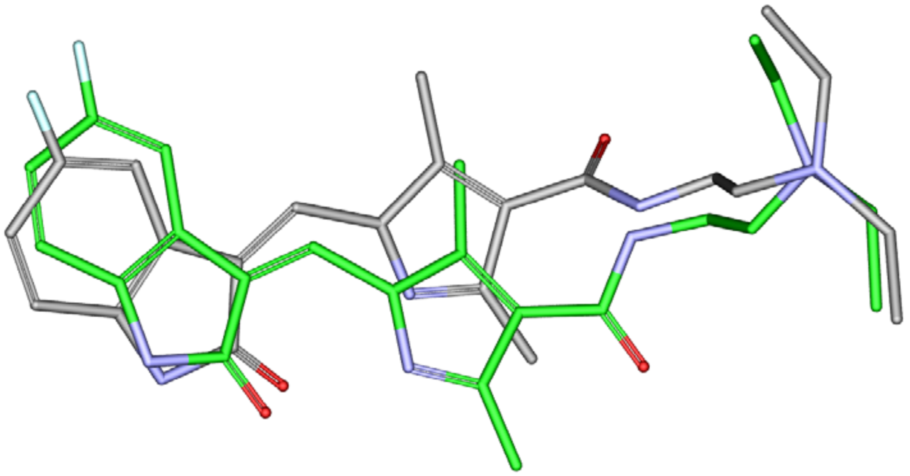

Figure S8. Aligned structures of the original ligand conformation (green) present in 2Y7J and the docked ligand conformation (gray) of sunitinib in 2Y7J crystal structure. RMSD = 1.50Å, calculation done using Discovery studio 2.1.

Table S1. The top 97 compounds ranked by DOCK6.0/LigFit score and thier percent FLT3 inhibition at 10μM.

Corporate ID		MW	Dock / Ligfit score	Inhibition %	Corporate ID		MW	Dock/ Ligfit Score	Inhibition %
BPR001(CSV0A031467)	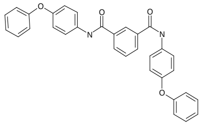	500.559	-372/110	-0.90%	BPR050(CSV0A062560)	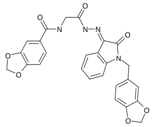	500.471	-356/104	0.40%
BPR002(CSV0A011296)	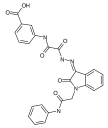	485.46	-369/107	1.70%	BPR051(CSV0C001836)	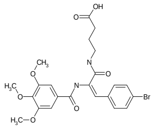	521.368	-356/96	0.90%
BPR003(CSV0A042562)	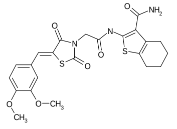	501.584	-369/101	0.50%	BPR052(CSV0C008541)	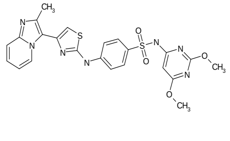	523.596	-356/109	6.00%
BPR004(CSV0A048785)	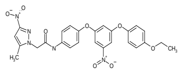	533.501	-369/108	0.80%	BPR053(CSV0A008714)	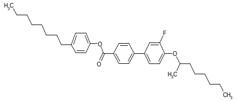	532.745	-355/108	-2.20%
BPR005(CSV0A013828)	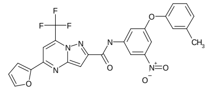	523.431	-368/108	-2.50%	BPR054(CSV0A013730)	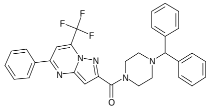	541.58	-355/105	-0.20%
BPR006(CSV0A014367)	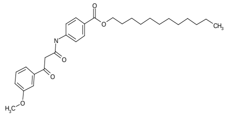	481.637	-368/100	-2.70%	BPR055(CSV0A031501)	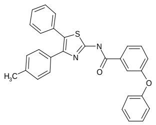	462.574	-355/100	-1.00%

BPR007(CSV0A051509)	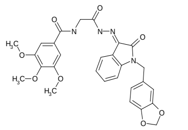	546.541	-368/108	2.80%	**BPR056(CSV0A034160)**	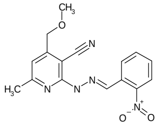	**325.33**	-355/91	**82.10%**
BPR008(CSV0A005771)	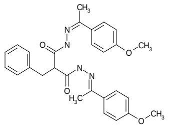	486.575	-367/98	0.10%	BPR057(CSV0A034206)	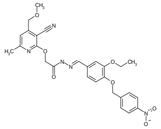	533.545	-355/106	0.90%
BPR009(CSV0A035490)	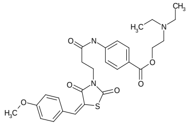	525.628	-367/102	6.60%	BPR058(CSV0A034595)	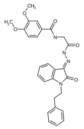	486.532	-355/103	2.60%
BPR010(CSV0A038176)	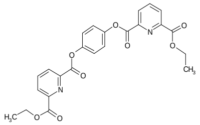	464.435	-367/101	2.50%	BPR059(CSV0A043513)	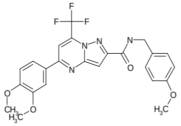	486.454	-355/104	0.80%
BPR011(CSV0A029882)	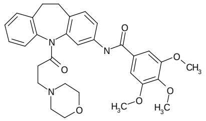	545.641	-366/107	7.60%	BPR060(CSV0A051510)	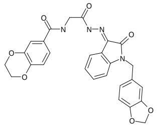	514.498	-355/107	0.90%
BPR012(CSV0A041534)	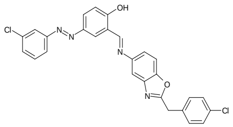	501.376	-366/104	2.10%	BPR061(CSV0A059600)	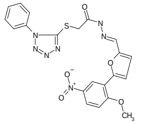	479.477	-355/102	4.90%
BPR013(CSV0A008382)	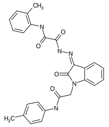	469.504	-365/103	0.00%	BPR062(CSV0A061707)	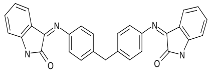	456.508	-355/108	0.80%

BPR014(CSV0A017765)	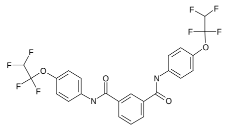	548.393	-365/102	0.70%	BPR063(CSV0C002699)	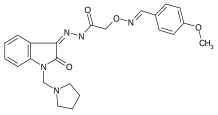	435.486	-355/101	0.30%
BPR015(CSV0A041988)	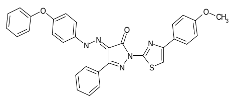	545.624	-365/112	-0.40%	BPR064(CSV0A028575)	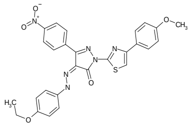	542.577	-354/111	2.00%
BPR016(CSV0A016908)	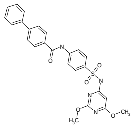	490.541	-364/103	0.50%	BPR065(CSV0A034602)	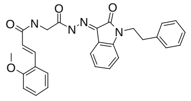	482.543	-354/108	1.10%
BPR017(CSV0A021846)	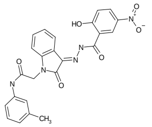	473.448	-363/112	1.50%	BPR066(CSV0A010719)	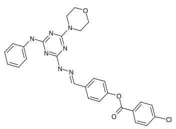	529.99	-353/105	0.10%
BPR018(CSV0A052457)	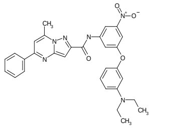	536.595	-363/111	3.50%	BPR067(CSV0A012867)	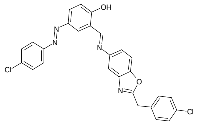	501.376	-353/103	2.00%
BPR019(CSV0A062557)	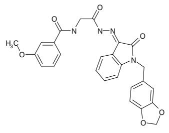	486.488	-363/104	1.30%	BPR068(CSV0A019739)	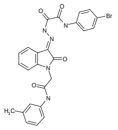	534.373	-353/106	-1.50%
BPR020(CSV0B012531)	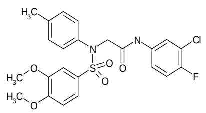	492.957	-363/94	0.70%	BPR069(CSV0A034333)	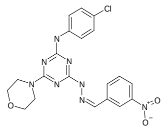	454.879	-353/99	1.60%

BPR021(CSV0A005350)	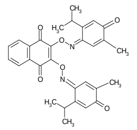	512.57	-362/100	13.20%	BPR070(CSV0A048895)	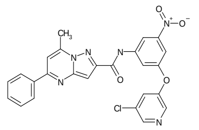	500.905	-353/103	2.30%
BPR022(CSV0A034868)	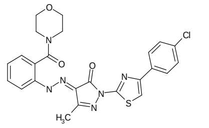	508.99	-362/98	-2.20%	BPR071(CSV0A061227)	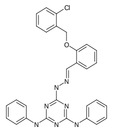	522.013	-353/111	1.40%
BPR023(CSV0A019616)	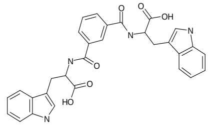	538.564	-361/113	0.90%	BPR072(CSV0A004079)	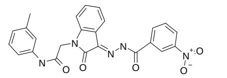	457.449	-352/101	0.70%
BPR024(CSV0A024759)	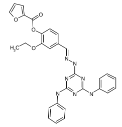	535.567	-361/114	0.60%	BPR073(CSV0A026290)	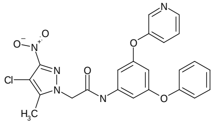	479.883	-352/103	0.10%
BPR025(CSV0A032833)	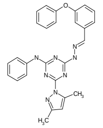	476.545	-361/111	0.50%	BPR074(CSV0A030936)	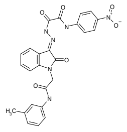	500.474	-352/109	0.20%
BPR026(CSV0A034205)	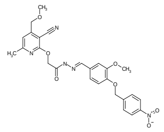	519.518	-361/107	1.50%	BPR075(CSV0A045095)	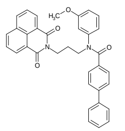	540.624	-352/111	1.30%
BPR027(CSV0C001841)	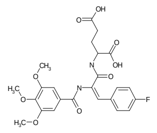	504.473	-361/103	-0.20%	BPR076(CSV0A053019)	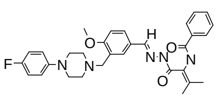	543.646	-352/118	1.10%

BPR028(CSV0A009104)	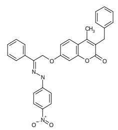	519.562	-360/109	-0.60%	BPR077(CSV0A055635)	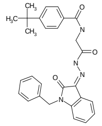	468.56	-352/112	4.80%
BPR029(CSV0A043963)	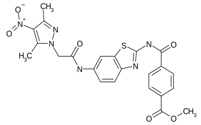	508.516	-360/101	0.90%	BPR078(CSV0A043498)	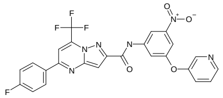	538.421	-351/116	1.30%
BPR030(CSV0B003578)	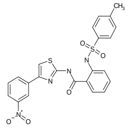	494.551	-360/102	0.30%	BPR079(CSV0A015737)	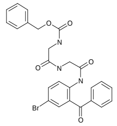	524.375	-350/97	0.70%
BPR031(CSV0B028719)	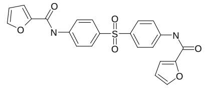	436.446	-360/92	1.70%	**BPR080(CSV0A027968)**	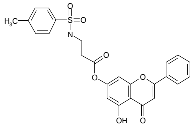	**479.512**	-350/105	**41.60%**
BPR032(CSV0C002695)	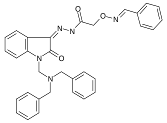	531.619	-360/117	2.50%	BPR081(CSV0A034159)	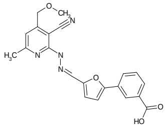	390.4	-350/89	22.80%
BPR033(CSV0C002703)	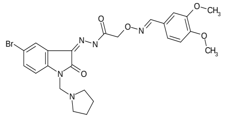	544.409	-360/96	3.10%	BPR082(CSV0A034596)	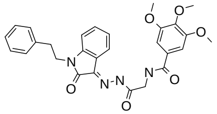	516.558	-350/104	1.00%
BPR034(CSV0C012196)	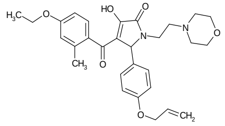	506.604	-360/105	0.90%	BPR083(CSV0A034609)	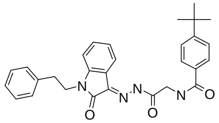	482.587	-350/104	3.00%

BPR035(CSV0A038787)	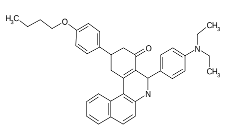	544.743	-359/114	1.50%	BPR084(CSV0A043957)	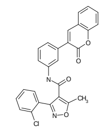	456.889	-350/96	2.90%
BPR036(CSV0A053548)	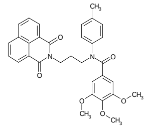	538.605	-359/114	4.20%	BPR085(CSV0A056184)	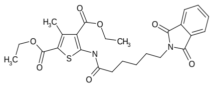	500.575	-350/106	1.00%
BPR037(CSV0A062559)	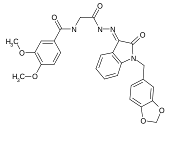	516.514	-359/114	4.90%	BPR086(CSV0A058884)	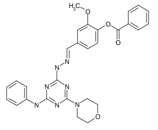	525.571	-350/108	0.90%
BPR038(CSV0A029878)	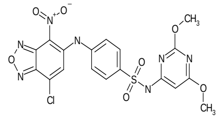	507.871	-358/98	1.70%	BPR087(CSV0A000015)	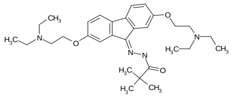	508.71	-349/101	-0.60%
BPR039(CSV0A043865)	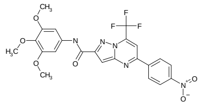	517.425	-358/99	1.70%	BPR088(CSV0A019152)	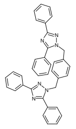	544.664	-349/118	0.10%
BPR040(CSV0A059009)	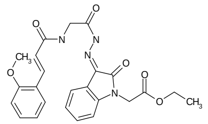	464.482	-358/102	2.40%	BPR089(CSV0A024460)	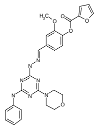	515.533	-349/104	0.90%
BPR041(CSV0A022916)	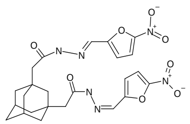	526.51	-357/104	0.70%	BPR090(CSV0A028568)	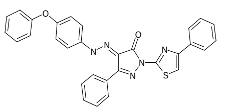	515.598	-349/111	-0.20%

BPR042(CSV0A030020)	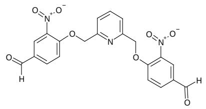	437.369	-357/101	-0.60%	BPR091(CSV0A034045)	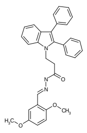	503.606	-349/110	0.30%
BPR043(CSV0A031741)	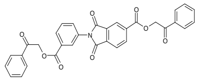	547.526	-357/119	0.60%	BPR092(CSV0A036372)	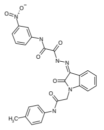	500.474	-349/109	0.40%
BPR044(CSV0A036448)	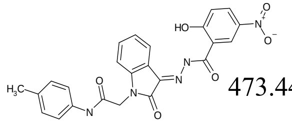	473.448	-357/113	2.40%	BPR093(CSV0A039930)	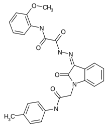	485.503	-349/105	1.10%
BPR045(CSV0A048961)	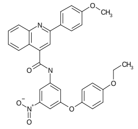	535.56	-357/116	3.50%	BPR094(CSV0A051514)	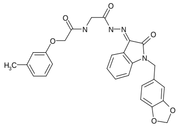	500.515	-349/109	2.20%
BPR046(CSV0A051508)	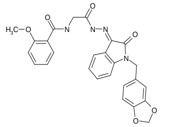	486.488	-357/105	2.90%	BPR095(CSV0C002702)	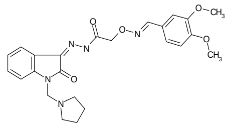	465.513	-349/97	0.20%
BPR047(CSV0B026256)	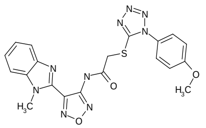	463.48	-357/97	-0.70%	BPR096(CSV0C002898)	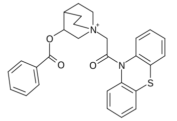	471.602	-349/97	0.40%
BPR048(CSV0C008368)	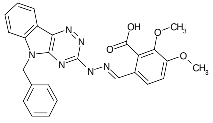	482.503	-357/110	10.90%	BPR097(CSV0C014232)	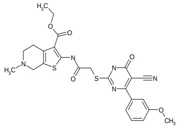	539.636	-349/102	3.10%

BPR049(CSV0A000009)	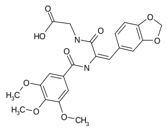	458.428	-356/102	0.10%	Sunitinb	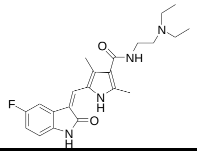	398.484	-300/89

**Assay control compound**	**2 µM**	**0.1 µM**	**IC$_{50}$ (µM)**
Sorafenib	82.10%	45.20%	0.102

Table S2. Comparison of enrichment factor (EF) of our study with 5 recently reported virtual screening experiments

**Comparison**	**Our model**	**J Chem Inf Model. 2011, (51): 755.**	**J Chem Inf Model. 2013 (53),809**	**ChemMedChem. 2014, (9): 953.**	**J Mol Graph Model. 2014, (53): 31**	**Bioorg Med Chem Lett. 2014 (5): 1261.**
**Screen method**	**Homology model+Structure-based VS**	**Homology model+Structure-based VS**	**Ligand-based+Structure-based VS**	**Ligand-based VS**	**Ligand-based VS**	**Structure-based VS**
Total number of active molecules identified (hits identified)	2	3	1	7	5	2
Total number of molecules selected (no. selected)	97	25	24	151	357	50
Total number of active molecules (hits total)	2	3	1	7	5	2
Total number of molecules in database (no. Total)	125000	1125	77931	125000	200000	4000
Hit IC_{50} range	2.3-10.7 μM	2-58 μM	14.4 μM	1.29-11.71 μM	0.83-1.12 μM	4.05-5.54 μM
Hit rate[a]	2.06	12	4.7	4.64	1.40	4
Enrichment factor (EF)[b]	1288.66	45.00	3247.13	827.81	560.22	80.00

[a]Hit rate =(hits identified /no. selected); [b]EF = (hits identified /no. selected)/ (hits total/no. total)

Table S3: Docking parameters used for the study

Docking parameters			
calculate_rmsd	yes	minimize_ligand	yes
use_rmsd_reference_mol	yes	minimize_anchor	yes
orient_ligand	yes	minimize_flexible_growth	yes
automated_matching	yes	use_advanced_simplex_parameters	yes
max_orientations	3333	simplex_anchor_max_iterations	10
critical_points	yes	simplex_anchor_max_cycles	1
chemical_matching	yes	simplex_anchor_score_converge	0.1
use_ligand_spheres	no	simplex_anchor_cycle_converge	0.1
flexible_ligand	yes	simplex_anchor_trans_step	0.1
min_anchor_size	50	simplex_anchor_rot_step	0.1
pruning_use_clustering	yes	simplex_anchor_tors_step	10
pruning_max_orients	10	simplex_grow_max_iterations	10
pruning_clustering_cutoff	10	simplex_grow_max_cycles	3
use_internal_energy	yes	simplex_grow_score_converge	0.1
internal_energy_att_exp	6	simplex_grow_cycle_converge	1
internal_energy_rep_exp	12	simplex_grow_trans_step	1
internal_energy_dielectric	4	simplex_grow_rot_step	0.1
use_clash_overlap	yes	simplex_grow_tors_step	50
clash_overlap	0.3	simplex_final_min	yes
bump_filter	yes	simplex_final_min_rep_rad_scale	1
max_bumps_anchor	12	simplex_final_min_add_internal	yes
max_bumps_growth	8	simplex_final_max_iterations	40
score_molecules	yes	simplex_final_max_cycles	1
contact_score_primary	yes	simplex_final_score_converge	0.1
contact_score_secondary	no	simplex_final_cycle_converge	1
contact_score_cutoff_distance	4.5	simplex_final_trans_step	1
contact_score_clash_overlap	0.5	simplex_final_rot_step	0.1
contact_score_clash_penalty	10	simplex_final_tors_step	50
grid_score_secondary	yes	simplex_secondary_minimize_pose	no
grid_score_rep_rad_scale	1	simplex_random_seed	0
grid_score_vdw_scale	1	atom_model	all
grid_score_es_scale	1		
